# Nutritional and Functional Properties of Colostrum in Puppies and Kittens

**DOI:** 10.3390/ani11113260

**Published:** 2021-11-15

**Authors:** Luciana Rossi, Ana Elena Valdez Lumbreras, Simona Vagni, Matteo Dell’Anno, Valentino Bontempo

**Affiliations:** Department of Health, Animal Science and Food Safety “Carlo Cantoni”-VESPA, Università degli Studi di Milano, 26900 Lodi, Italy; anaelenavaldez@hotmail.com (A.E.V.L.); simona.vagni@gmail.com (S.V.); matteo.dellanno@unimi.it (M.D.); valentino.bontempo@unimi.it (V.B.)

**Keywords:** nutrition, nutraceutical, puppies, kittens, neonatology, milk, colostrum

## Abstract

**Simple Summary:**

The post-natal period is a crucial time for all animal species. During the course of their first two weeks of life, puppies and kittens face several risks to their health due to their scarce energy storage and weak immune system. Colostrum is the first production of the mammary glands that plays a pivotal role for puppies and kittens. Colostrum is an important source of immunoglobulins and key nutrients such as lipids and carbohydrates, which are fundamental for the health of newborns. Puppies and kittens must ingest a sufficient amount of colostrum within a few hours of birth to ensure their survival. On the other hand, there are some particular compounds that are not strictly essential, but their presence may play an important role in nutrition and health. As there are no recent studies on companion animals, we have reported published articles describing animal studies in different species to review the nutrition of newborn mammals, with particular emphasis on companion animals.

**Abstract:**

The present review aims toward a better understanding of the nutrition of newborn puppies and kittens. The post-natal period is very sensitive in dogs and cats, as in other animal species. During the first two weeks of life, puppies and kittens are at high risk of dehydration, hypothermia, and hypoglycemia, as well as infectious diseases as they start to acquire the physiological functions of the adult. Neonatal hepatic glycogen storage is low, and newborns depend on colostrum intake to survive. Colostrum provides immunoglobulins and other important substances such as lipids and carbohydrates. Immunoglobulins are central to the immunological link that occurs when the mother transfers passive immunity. The mechanism of transfer varies among mammalian species, but in this review, we focused our attention on dogs and cats. Furthermore, there are components of colostrum which, although their presence is not absolutely necessary, play an important role in nutrition. These components have received considerable interest because of their presumed safety and potential nutritional and therapeutic effects both in humans and animals; however, unfortunately, there are few recent studies in companion animals. Here, we have gathered the published articles that describe studies involving different species of animals, emphasizing companion animals. In particular, the purpose of this narrative of the nutritional and functional proprieties of queens’ and bitches’ colostrum.

## 1. Introduction

The neonatal period of dog and cat, a short transition phase usually defined as the first two weeks of life, is a very crucial time because of the adjustment to the extrauterine environment and the preparation for relatively greater independence post-weaning [1]. In this phase, nutrition plays a pivotal role, not only for relapses on health during the early stages of life, but particularly for its long-term effects. Indeed, inadequate nutrition or an inability of the gastrointestinal tract to digest and absorb nutrients can affect growth and health status or cause, in worst-case scenarios, premature deaths. Moreover, experimental evidence indicates that feeding is an important factor in socialization, and lactation represents a critical window for body weight and food intake programming in different species [2,3,4,5]. 

Firstly, an efficacious nutritional strategy includes correct management of bitches and queens during both gestation and lactation, in order to ensure the right fetal development and optimize colostrum and milk production [6,7]. Canine and feline colostrum is defined as the first secretion of the mammary glands during the first two days postpartum [6]. Puppies and kittens satisfy their nutritional requirements through colostrum consumption, for which quality (immunoglobulin level) is of fundamental importance. Even if the mother’s colostrum cannot be fully substituted by other products, in case of its absence or deficiencies, milk replacers must be used in order to meet the nutrient requirements of the newborn [8]. Therefore, awareness of the role that this period plays in the health status of adults could promote a better understanding of the nutritional problems of newborns that, in practice, may be undervalued or not clinically handled with proper care. Even if colostrum composition is widely studied in other animal species, only few papers are available for canine and feline species. This paper aims to provide a narrative review of the nutritional and functional proprieties of queens’ and bitches’ colostrum. In particular, the purpose of this paper is to underline the importance of a correct nutritional management of puppies and kittens in light of recent scientific insights on the role of bioactive components of colostrum on health status.

## 2. Development of the Gastrointestinal System and Food Inputs

In the development of the mammalian digestive tract, many phases have been identified from embryonic organogenesis to weaning [9]. At birth, although the gastrointestinal system is completely formed from a structural viewpoint, it is still immature and during the post-natal period, a gradual increase in digestive function occurs. The first weeks of life are a critical period for the animals because of the physiological immaturity of different systems, in particular the gastrointestinal tract. Indeed, in the first 24 h after birth, the canine small intestine nearly doubles in weight [10].

The first compound to enter the gastrointestinal system is the amniotic fluid, an important bioactive buffer that plays a significant role in gut development and serves as a barrier to the outside world [9]. The gut represents an important system that exerts selective digestive and absorptive functions, changing constantly in response to body requirements and the outside environment. The two most important functions of the gut are, firstly, the digestion and absorption of essential nutrients and secondly, the physical and immunological barrier function against pathogens and toxins through specific and nonspecific defenses. Changes in size and morphology of the gut, including proliferation of enterocytes, are also influenced by different characteristics of food and other ingested substances [11,12]. Colostrum intake could also significantly impact the long-term development of a newborn after weaning through the stimulation of organ development, particularly of the gastrointestinal tract [13].

## 3. Composition and Physiological Properties of Colostrum

Colostrum represents the secretion of the mammary gland at the time of the final third of pregnancy. Its major nutrients are lipids and carbohydrates that are their main source of energy. In colostrum, the protein fraction is mainly constituted by casein and immunoglobulins, whose role is fundamental for passive immune transfer [14]. During the newborn life, colostrum provides essential nutrients such as water, growth factors, digestive enzymes, and maternal immunoglobulins (Table 1). Meconium is constituted by the first feces of a newborn, and it is composed of the ingested materials from the fetal life in the uterus [15]. Colostrum plays several significant roles for the newborn: it helps the elimination of meconium by its laxative action, its useful nutrient content limits hypoglycemia and begins the extrauterine growth, it protects against hypothermia by supplying energy, and provides systemic transfer of immunity and digestive local immunity [16]. Moreover, many recent studies focus their attention on the multiple functional properties of colostrum related to immune support and regenerative effects that extend to all structural body cells, such as the gut [17,18,19].

### 3.1. Nutritional Function and Composition

The exact colostrum composition of carnivores is not extensively known, and data reported in the literature are characterized by high variability [23]. Table 2 shows the concentration of nutrients during the first week of lactation of bitches and queens. Considering calories, the colostrum energy is nearly equally provided by proteins (50% of colostral calories) and by lipids (40% of colostral calories), and the energy concentration (kcal/mL) is similar in colostrum and in milk, but the pattern during lactation differs slightly between bitches and queens [6]. In dogs, the energy value progressively decreases by 20% during the two first weeks postpartum, whereas in queens, it rapidly drops (−30% over the first 3 days) and then increases progressively over the whole lactation period [22,23,24]. Considering carbohydrates, lactose represents, as in other animal species, the principal sugar source, although its concentration in bitch and cat milk is about 30% lower if compared to cows’ milk. The lactose concentration in queen and bitch colostrum was 29.9 g/L and 16.6 g/L, and approximately 35–42 g/L in mature milk [22,23]. Lactose is the only glucose source for a puppy during the first month of life, as the pancreatic activity of amylase is practically nonexistent and, therefore, other carbohydrates cannot be digested. The digestion of lactose remains constant up to two months of age, and then, there is a sharp decline in intestinal lactase. This sharp decline is responsible for outbreaks of diarrhea in patients over 2–3 months of age who are given a large amount of milk [25]. Vitamin E is required to protect the newborn against oxidative stress and vitamin A is fundamental for growth and development. Furthermore, colostrum of livestock animals was found to contain higher concentrations (approximately 80% more in cows’ milk) of vitamins A and E compared to mature milk [24]. Vitamin A is essential for cellular differentiation, vision, skin health and protein synthesis. A lack of vitamin A causes depression of immune function, mainly cell-mediated function, and alteration of nonspecific defenses. Vitamin A deficiency can lead to metaplasia and keratinization in skin and mucous membranes [26]. Vitamin D is essential for blood calcium regulation through intestinal absorption, which is fundamental for bone development, and is also implicated in a properly functioning immune system. Vitamin D must be supplemented with the diet since puppies and kittens cannot convert dehydrocholesterol in the skin to a more active form of vitamin D [27]. In addition to calcium absorption, in the intestine, vitamin D also acts on phosphate absorption, influencing bone growth, mineralization and remodeling [28]. Calcium-phosphorus ratio is generally stable over time in a bitch’s colostrum and milk (1.5) and rises from colostrum to milk in queens [23]. The micronutrient content of colostrum strongly contributes to puppies’ and kittens’ health and growth.

### 3.2. Immune Function

Colostrum contains approximately a double concentration of proteins compared to milk, and most of these (20–30%) are represented by immunoglobulin (IgG, IgM, IgA), which provide systemic and local immunization of the newborn. Immunoglobulin intake is vital for carnivore neonates as it provides partial humoral protection to complement the limited placental transfer of immunoglobulins during gestation [14]. Carnivore species have an endotheliochorial placenta in which there are four cell layers (comprising maternal endothelium and the chorionic epithelium), allowing the passage of approximately 10–20% of the mother’s IgG [29]. Generally, it is accepted that only 10% of the maternal circulating antibodies pass by the transplacental way in the carnivores, such that the newborn puppy or kitten has a serum IgG concentration of approximately 5% compared to the adult level [30]. Colostrum is particularly rich in IgG and also contains IgA and IgM antibodies that are produced locally in the mammary tissue [6,31]. Total IgG concentration is about 20–30 g/L in bitch colostrum and 50–70 g/L in queen colostrum [6], but they drastically decrease, especially during the first 24 h, so colostrum must be ingested as soon as possible (within the first 16 h) after birth by newborn puppies and kittens to fulfill its immunizing role. For this reason, the absence of colostrum intake can cause several disorders and impairs immune system development [32]. 

For dogs, 10–20% of IgG transferred from the mother to the newborn is via the placenta; the remainder is supplied by the colostrum [33]. By this passive transfer, immunoglobulins can pass the intestinal barrier of newborn animals. According to Poffenbarger et al. [33], the maximum effectiveness of absorption of colostral immunoglobulins occurs eight hours after birth and needs to be completed within 16–24 h [34]. Other authors showed that immunoglobulins cannot pass the digestive barrier beyond a 15 h deadline [35] or after the first 24 h of life [32]. After this time, the gut epithelium becomes impermeable preventing immunoglobulin absorption [25]. 

In the cat, immunity transfer must be completed within 12 h postpartum to obtain a maximum rate of immunoglobulin serum in the kitten. Absorption becomes impossible between 16 and 36 h of life. During normal development, absorption of undigested proteins occurs until the concentration of the trypsin inhibitors of colostrum decreases and protein digestion begins, namely toward 24–36 h of life in the cat [36]. Rapid ingestion is necessary not only due to the reduction in immunoglobulin concentration in mammary secretions, but also because of the decrease in its intestinal absorption. Colostral immunoglobulins are transferred to the blood by the small intestine [37]. Within the 24 h after birth, in the intestines of puppies, there is a nonselective macromolecular transport which allows the transfer of immunoglobulins and other proteins from the intestinal lumen to the bloodstream without degradation. Once this absorption period is completed, humoral immunity is acquired [38]. Furthermore, newborns are characterized by a low production of pancreatic enzymes, which facilitates the absorption of intact immunoglobulins from colostrum. Macromolecule absorption capacity is related to the presence of intracellular vacuoles, less numerous in cats than in dogs [39]. The ability of intestinal absorption, experimentally evaluated by polyvinylpyrrolidone (PVP) supplementation, resulted lower in kittens than puppies. This was also reflected by a lower concentration of serum antibodies. PVP has non-selective macromolecular absorption. It is used as a model protein, as its absorption occurs from 10 to 14 days in kittens [39]. Physiologically, the characteristics of eosinophilic droplets absorption of undigested proteins are histologically visible in kittens up to 36 h of life and the antitrypsin activity of colostrum leads to the maintenance of these droplets for up to five days. Therefore, enterocytes retain their ability to absorb larger proteins if enzymatic digestion is inhibited [40]. A recent work in dogs, instead, highlighted that the absorption ability is reduced by 50% at 4 h after birth, although the absorption continues over 48 h [34].

### 3.3. Absorption Efficiency

Other than nutritive and immune functions, colostrum has a mild laxative effect on the newborn, encouraging the passage of meconium, which aids in the excretion of excess bilirubin and prevents jaundice [41,42]. The absorption of colostrum immediately after birth may play an important role in the establishment of postnatal circulatory volume [43]. 

The newborn without immunoglobulins acquires a passive systemic immunity due to colostrum intake during the first hours of life. The intestinal barrier of puppies and kittens is immature at birth. In particular, the tight junctions between enterocytes are not well developed and macromolecules such as immunoglobulins can pass through the intestinal epithelium within the first 12–16 h of life [14]. 

The loss of absorption efficiency is faster in puppies (12 h) than in kittens where it happens approximately at 16 h of life [34,44]. Nevertheless, the permeability of the digestive mucous membrane during the first hours of life allows the absorption of many colostrum compounds [35]. The acquisition of colostral immunity during the first 24 h occurs thanks to a selective transport, which is carried out by the endocytosis of immunoglobulins after joining the receptors on the apical membrane of cells [45]. Therefore, immunoglobulins cross the basal membrane and access the blood circulation [39]. Thus, the enterocytes have a specific receptor for the transport of IgG. The neonatal γ-immunoglobulin (IgG) Fc receptor (FcγRn) transports, in a selective way, the Fc portion of IgG from the intestinal lumen to the circulation, avoiding their degradation [38]. This receptor has homologies with the antigens of major histocompatibility complex class I without having functional peptide binding grooves [46,47].

### 3.4. Other Bioactive Compounds

Colostrum also contains bioactive proteins and peptides that improve local immune defense (Table 3). Growth factors and hormones present in the colostrum play a crucial role in the development and health of young animals [48]. Growth factors can be transferred from the digestive tract toward the circulation and involve tissue development in other parts of the organism. For example, insulin-like growth factors (IGF) persist in the digestive tract and have local effects on cell growth [49]. These factors are generally found to be more concentrated in bovine colostrum and are likely biologically important for the survival of newborns and help the maturation of the digestive tract, as suggested in other livestock [50]. Several studies have clarified the role of these compounds, showing that during the transition to milk feeding, the development of the small intestine undergoes a noticeable decline [51,52,53]. This decline should not be interpreted as a stop, as even so-called mature milk (day 21 of lactation), although less so, has a trophic effect on the small intestine [54]. Indeed, milk possesses several bioactive substances including immunomodulatory, anti-inflammatory, and antimicrobial agents.

Even if the available studies have been conducted in ruminants, the nutraceutical proprieties of colostrum could be translated to other animal species. Colostrum is characterized by a high content of different growth factors and bioactive molecules that positively influence health status [67]. Trophic factors in mammalian colostrum promote the growth of the small intestine of neonates. Colostrum enhances the maturation decline in lactase activity and the expression of sucrose activity [68]. Small peptides and proteins of the innate immune system are increasingly valued for their potential as antimicrobic and immunomodulators [69,70]. Colostrinin (CLN), a proline-rich polypeptide complex, originally isolated from ovine colostrum, has been studied as a nontoxic natural preparation for the prevention of Alzheimer’s disease (AD), observing encouraging results [71]. 

The lactose concentration in canine and feline colostrum is low compared to milk, about 1.5% and 3.0%, respectively. Mammalian milk and colostrum usually contain, in addition to lactose, a plethora of neutral and acid oligosaccharides [72]. Oligosaccharides play an important role, as they may inhibit the adhesion of pathogenic microorganisms to the intestinal and urinary tract by acting as receptor analogs, thus preventing gastric and urinary infections [73,74]. In addition, milk oligosaccharides may act as prebiotics, promoting the growth of beneficial microorganisms, such as *Bifidobacterium bifidum*, within the lower gastrointestinal tract, and inhibiting pathogenic organism proliferation [75]. The immunomodulatory effects of livestock animal colostrum in humans have been demonstrated in several studies, including infectious diseases [76,77], exercise-induced immune suppression [78,79], wound healing involving gastrointestinal damage [53,80,81] and bone density [82]. This has led to several investments in antimicrobial molecules that are currently at various development stages by biotechnology companies [83]. With the use of milk as a food on one side, and the development of novel drugs based on isolated colostrum compounds on the other, the nutraceutical use of colostrum extracts in health management is an expanding niche [84] and is receiving interest as a complement to or substitutes for vaccines and pharmaceutical drugs [85,86]. Despite the commercial application of colostrum and colostrum by-products, it represents an indispensable nutraceutical for newborns. In polytocous species, those bearing multiple offspring in a litter, such as dogs and cats, the degree of passive immunization is often variable within the same litter due to colostrum absorption variability. Indeed, the last born could potentially ingest an insufficient amount of colostral immunoglobulins, and this represents an important risk for pathology development [87].

## 4. Colostrum Deficiencies: Causes and Consequences

**Figure 1:**
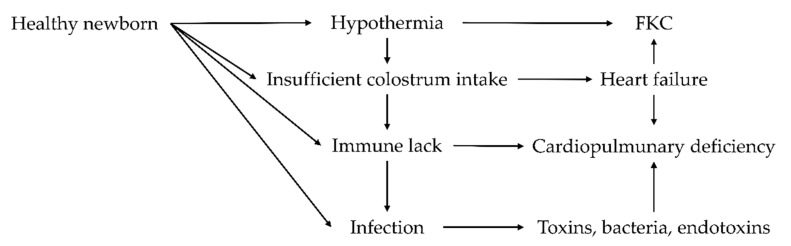
Causes and interactions of Fading Kitten Complex (FKC) or Fading Puppy Complex (FPC) [92,93].

The causes of colostrum absence may be due to several factors related to the mother or the newborns, and they are similar in dogs and cats (Figure 1). Some of these factors are related to the colostrum and milk production of the mother [88]. In general, primiparous females are more stressed, since they face a birth for the first time, than multiparous. In addition, systemic diseases, dystocia, or local inflammation can decrease the quantity and quality of mammary production and the attitude to breastfeeding [89]. Agalactia can be induced by colostrum deprivation of newborns due to the risk of isoerythrolysis development. Other factors influencing agalactia could be related to the environment, habits or other intrinsic factors of the newborn [35]. Weakness or hypothermia in newborns incapable of breastfeeding is often associated with a malformation that prevents breastfeeding (cleft palate) [90]. Kittens have a higher risk of isoerythrolysis compared to puppies [91]. An important characteristic of the feline blood group system is the presence of naturally occurring alloantibodies against the blood type that they lack. Natural means that there is no need for previous exposition to blood or blood products [91]. The clinical signs of this pathology are similar to those of neonatal sepsis, so it is difficult to recognize. After birth, the newborn enters a persistent status of hypothermia, and it loses weight rapidly. During isoerythrolysis, heart rate and breathing slow, and episodes of apnea occur. Vocalizations and bronchial rales are typical signs of this issue. Subsequently, hyperextension of the spine and limbs appears. During the final steps of isoerythrolysis, breathing stops, and then, cardiac death occurs [92].

### 4.1. Cardiovascular Troubles

The volume of colostrum ingested after birth contributes to the supply of liquid for the newborn circulation. An insufficient fluid intake is the cause of circulatory disorders [35]. Immaturity of the cardiovascular system of the puppy during the first five days of life prevents it from combating multiple stressors. For this reason, the contribution of liquid and the production of colostrum by the mother must be able to maintain the blood volume. Insufficient fluid intake may cause a significant decrease in blood volume resulting in fatal heart failure [35]. In addition, the turnover of water in puppies is very high during the neonatal period. It is approximately double that of adults. This is due to an immature kidney function making them susceptible to dehydration. The fluid maintenance requirement for neonatal puppies is approximately 132–220 mL/kg/day [94]. 

Insufficient colostrum intake could represent the origin of circulatory problems. The baroreceptors are not functional until the fourth day of life, and heart rate variability, as in adults, depends on the blood pressure. The activity of the inotropic agents (atropine, dopamine, dobutamine) becomes normal only toward 9–10 weeks of life [31]. 

The heart rate of puppies normally ranges from 180 to 250 beats per minute. The event of hypoxia decreases in puppies and kittens compared to adults [95]. The blood pressure reaches the adult values only around six weeks of life. It varies depending on many factors, including core temperature and blood glycemia: colostrum lacking could lead to severe a drop of heart rate [96]. 

### 4.2. Hypothermia

There are several factors that act in the maintenance of thermogenesis (production of heat) and thermolysis (loss of heat) after birth. Rectal temperature varies with age and environment [25]. Puppies’ and kittens’ rectal temperature normally declines after birth, possibly as an adaptation mechanism to protect from hypoxia and acidosis by reducing metabolic demand [1]. Newborns can dissipate heat by several processes (evaporation, conduction, radiation, convection); this loss is more intense as the body surface is largely proportional to the volume. These losses can be increased if the environment is cold or if the newborn is separated from its mother [25]. During the first week of life, brown fat is the main source of thermogenesis. After this period, the shivering reflex permits thermoregulation by the newborn itself. The difficulties encountered by the newborn related to thermoregulation could be classified into three critical points: absence of the shivering reflex and mechanisms of vasoconstriction up to six days and the low presence of fat in the hypodermic system [97]. The normal rectal temperature is between 35 and 37 °C for the newborn [98]. The rectal temperature gradually rises to adult levels by the age of seven weeks. In addition, hypoxia involves a context of anaerobiosis limiting the possibility of compensating by oxidations and thermolysis [99]. The newborn gives a behavioral answer to the heat loss: it seeks an external source of heat (e.g., udder of the mother, contact of its brothers and sisters). The drying of newborns is crucial for the management of their temperature [98]. The absorption of colostrum contributes with its nutrients to support thermogenesis. The liver immediately exploits carbohydrates, then fats and other components for energy production [100]. The difficulties of temperature control thus play a crucial role in the etiology of neonatal mortality. Indeed, a premature birth or incorrect management can compromise homeothermy by increased thermolysis that leads to higher energy consumption for thermogenesis [25].

### 4.3. Hypoxia, Anoxia and Acidosis

As a consequence of insufficient colostrum intake or its deficiency, hypoxia, acidosis and anoxia may occur in newborns. Hypoxic animals may not show a hyperventilation stage, thus complicating diagnosis, although it is normally followed by late respiratory depression [1]. A further complication is combined with respiratory and metabolic acidosis that is a normal event after canine and feline birth [101]. Hypoxia at birth also can predispose to fatal necrotic enterocolitis [1]. Anoxia is associated with bradycardia and hyperventilation in dogs and cats before the fifth day of age, and is often followed by hypothermia and subsequent lower oxygen demand [102].

### 4.4. Hypoglycemia

The fetus receives a continuous infusion of glucose from the placenta, so it does not depend on its own gluconeogenesis. Due to their low fat and glycogen storages at birth, puppies may develop hypoglycemia in case of insufficient colostrum intake. During starvation, gluconeogenesis becomes the sole means of glucose homeostasis. The neonate’s small muscle mass decreases the use of free fatty acids as an alternative energy source and a possible lack of gluconeogenesis enzymes limits the neonate’s ability to maintain normal glucose levels [25].

## 5. Supporting Newborns in the Case of Colostrum Deficiencies

Companion animal survival in the newborn stage of life is highly influenced by colostrum intake and quality as it possesses many functional properties that guarantee an animal’s health status. When colostrum quality is poor (less than 20 g/L of IgG for bitch and less than 50 g/L of IgG for queen colostrum [6]), many clinical signs and symptoms must be recognized and treated. Important systemic biochemical changes occur when neonates are suffering, often related to multiorgan failure: hypoglycemia, hypovolemia, hypoalbuminemia, deficits of energy utilization, weight loss, altered acid–base balance. Hydration depends on a synergistic effect of environmental temperature and water loss rate. Moreover, approximately 80% of a newborn’s body weight is water; therefore, dehydration can occur quickly [103]. Dehydrated neonates may require from 60 to 180 mL/kg/day of fluid support [1]. Oral rehydration is preferred if gut functionality is normal, and the newborn is not hypothermic. Dehydrated animals have increased susceptibility to bacterial infections; parenteral routes for hydration need to be adopted with scrupulous sterile techniques [104]. Subcutaneous administration is the parenteral route most frequently adopted. If puppies lose more than 10% of weight during the first 24 h, administration of warm fluids is necessary to ensure puppy and kitten survival [105]. When treating a dehydrated puppy, fluid should be administered to meet the ongoing maintenance requirement (generally 60–100 mL/kg/day or 3–4 mL/kg/hour). It is important to highlight that a typical Labrador retriever neonate, weighing approximately 0.4 kg, will require 1.4 mL per hour as well as to replace the deficit. Oral electrolytes may be considered in mild cases only when bowel sounds are present. Generally, intravenous, intraperitoneal or intraosseous administration is required [106]. If a kitten is dehydrated, normothermic and not under shock or cardiovascular collapse, warmed subcutaneous fluids can be administered. The oral administration of warmed fluids can be considered if gastrointestinal troubles are absent [107]. If a kitten is moderately to severely dehydrated and large enough to facilitate intravenous therapy, this could be the most effective route. The cephalic or jugular vein can be catheterized and rehydrated with Ringer’s lactate solution, and from 1.25% to 5% dextrose can be added if necessary [103]. Warmed fluids may be given at 1 mL/30 g body weight (30 to 45 mL/kg), followed by a maintenance infusion phase of 80 to 120 mL/kg/d [108].

Hypoglycemia (<30–40 mg/dL) of a puppy requires slow intravenous dextrose administration from 0.5 to 1 g/kg from a 5–10% dextrose solution in Ringer’s lactate or normal saline, or by intravenous supplementation of 1–2 mL of 10–25% glucose to severely depressed puppies. Blood glucose concentration should then be monitored to evaluate hyperglycemia status [72,74]. In kittens, clinical hypoglycemia occurs when glucose level is <50 mg/dL and is a common issue for the newborn due to immature liver function and rapid depletion of glycogen [107]. In this case, administration of 5–10% of dextrose by gastric tube should be adopted [108]. Severe cases of kitten hypoglycemia should be treated by bolus infusion of 12.5% of dextrose intravenously or orally, followed by a constant-rate infusion of 1.25–5% dextrose in a balanced electrolyte solution to prevent rebound hypoglycemia [109]. The hematologic analysis may provide useful information. Normal values for puppies from birth to four weeks of age, along with adult values, can be found in standard textbooks [110,111]. It is important to keep accurate records when collecting blood samples from puppies and not to collect more than 10% of the animal’s circulating volume in one week, especially for repeated sampling for glucose level evaluation [112]. Treatment is continued until the resumption of feeding and a stabilized rectal temperature of 36 °C. If the maternal contribution is insufficient, a complementary artificial intervention is required. When the body temperature decreases to 35 °C, a solution of 10% glucose should be provided by orogastric route every 30 min until obtaining a rectal temperature of 37 °C. When a normal temperature is recorded, bottle feeding can then be restored. Due to the immaturity of the metabolic pathways of glucose regulation, iatrogenic hyperglycemia could be induced. Therefore, evaluation of the level of glycemia is suggested before carrying out new re-injections. In order to limit the number of newborns artificially nursed per litter, the prevention of hypoglycemia occurrence is necessary. Cold room temperature is associated with higher relative moisture that can induce respiratory distress [1]. The use of thermostat heating carpets, preferred compared to infrared lamps, heats the abdomen section and not the animal’s back. If the infrared lamp solution is chosen, it has to be placed at a sufficient distance to avoid excessive heating, burns, or dehydration. Hypothermia reversion requires slow warming, avoiding exceeding 1 °C per hour using heat pads wrapped in towels, heat lamps or bottles. Maintenance of the correct environmental temperature and a medium environmental humidity is required. It should not move below 55% (a value of 65% is considered ideal) [113]. Newborn warming could be carried out in an oxygen cage or incubator, representing a quick and safe alternative [107,114]. Puppies and kittens should not feed if their body temperature is lower than 34.4 °C and/or if bowel sounds cannot be heard. Providing dextrose at the same rate, as previously described, with any other fluid supplementation can help increase the calorific demand, which results from the rise in metabolic rate associated with reheating [106,111]. In case of colostrum deficiency, the colostrum bank could represent a good opportunity for the passive transfer of immunity to the litter. The bitch should be milked approximately 24 h after whelping; the colostrum should be frozen at −20 °C and warmed up to 30–35 °C before administration. It was demonstrated that freezing/thawing only slightly affects the immunity contained in colostrum [115]. Oral canine plasma supplementation (twice per day at two-day intervals from the second day of life until the fifty–sixth) was studied to possibly support puppies’ development, allowing higher weight gain, increased microbial diversity, and reduced risk of morbidity. Even if plasma administration showed to not directly improve the blood level of immunoglobulins, it demonstrated a beneficial effect on puppies’ health [116]. Another alternative strategy of immunization can be represented by the use of hyperimmune egg powder. Laying hens can be vaccinated against specific pathogens [117], and the hyperimmunized egg powder administered to puppies has been demonstrated to improve growth, fecal IgA and quality (score and pH), helping puppies to manage stress [118]. These strategies still require investigation in kittens.

## 6. Nutraceutical Compounds in Newborns

The growing interest of consumers in food quality has managed to establish new concepts such as the so-called “functional food”. Among them are nutraceutical substances, including those compounds that possess metabolites with pharmacological relevance, beneficial for human and animal health. Nutraceuticals can be defined as, “a food (or part of a food) that provides medical or health benefits, including the prevention and/or treatment of a disease” [119].

The veterinary medicine literature comprises several papers that investigated clinical and laboratory use of nutraceuticals.

We mainly focused our attention on three nutraceutical products that could be supplemented in milk replacers for puppies and kittens:

- Cow colostrum:

Cow colostrum is used as a nutraceutical treatment for animals of all ages to increase resistance to infections caused by a wide range of pathogens, including bacteria, viruses, parasites, and fungi. Cow colostrum has several components that are known to strengthen and enhance the immune system. In addition, it contains growth factors and bioactive compounds such as lactoferrin [120,121]. 

Lactoferrin is found in milk and mucosal secretions such as tears and saliva. Human colostrum showed the highest concentration, followed by cows’ milk. The supplementation of lactoferrin has been proposed due to its antibacterial and antiviral activities in the intestine, in part due to a direct effect on pathogens, but possibly also affecting mucosal immune function [122]. Lactoferrin has also been reported to enhance the iron levels of infants and pregnant women, possibly via the receptor-mediated pathway. In addition, lactoferrin can stimulate intestinal cells’ proliferation and differentiation.

- Polyunsaturated Fatty Acids (PUFAs).

PUFAs have a pivotal role in proper neonatal growth and development in all mammalian species. The canine retina is capable of synthesizing docosahexaenoic acid (DHA) from its precursor, docosapentaenoic acid (DPA). Puppies are able to accumulate DPA, but not DHA, in plasma phospholipids when the alpha-linolenic acid (ALA) precursor is fed [123]. It is likely, therefore, that the dog retina, and presumably other nervous tissues, synthesizes and utilizes DHA in a similar manner to other mammalian species and that plasma DPA provides the substrate for its synthesis [124]. Thus, a dietary source of preformed DHA or one of its precursors may be necessary during gestation and from parturition for a normal neural development in dogs and cats [125]. It is possible that ALA may be sufficient as a dietary precursor for the synthesis of required amounts of DHA during pre- and postnatal development. However, the requirement of ALA to optimize neural development in companion animals remains undefined. In addition, both n-6 and n-3 fatty acids precursors metabolically compete for the same enzyme systems. In light of this, their nutritional requirement is unclear [126]. 

DHA has gained considerable attention for its cardiovascular and anti-inflammatory effects. Research has revealed that one of the active metabolites of fish oil and flax seed oil is DHA. This compound is also a strong competitive inhibitor of cyclooxygenase, which is responsible for the conversion of arachidonic acid to the prostaglandins of two series. This is the main reason why omega-3 fatty acids exert their cardiovascular protective effects: the antithrombotic effects would be due to the reduction in thromboxane A2, prostaglandin G2, and prostaglandin H2, which are powerful inducers of platelet aggregation [127]. In addition to the DHA inhibition of prostaglandin synthesis, DHA reduces platelet responsiveness, thereby contributing to the antithrombotic effects of omega-3 fatty acids [128].

- Pre- and probiotics

Prebiotics and probiotics could interact with the immune composition of mammary secretions, influencing the distribution and relative abundance of specific immune responses in dogs and cats [129]; inducing higher luminal secretions of IgA in the small intestine; enhancing the immunoglobulins concentration in mammary secretions, by means of the immune entero-mammary link in monogastric species. As a consequence, higher levels of immunoglobulins could be transferred to puppies [130]. It was demonstrated that pre- and probiotics administration to the mother during pregnancy increases colostrum quality (higher immunoglobulin concentrations). Since colostrum plays a pivotal role in digestive immunity and the development of the gastrointestinal microbiota and of the digestive epithelium, puppies feeding on colostrum with a higher level of immunoglobulins will undergo better stimulation of the intestinal immune system [131]. It is important to highlight that the available studies on puppies and kittens have limitations similar to those for studies of adult dogs and cats, including small numbers of subjects, limited evaluations for disease states, and limited control populations for direct comparison. When puppies and kittens are concurrently evaluated, there are important species differences in response to probiotics; therefore, evidence from one species cannot automatically be extrapolated to another species. Probiotics are also likely to have different effects in immature animals than in adult animals as the gastrointestinal microbiota transitions to the adult microorganism population during development in immature animals [132].

## 7. Conclusions

The neonatal period is crucial for puppies’ and kittens’ survival as dramatic systemic changes may occur if their management is not properly approached. Colostrum administration is pivotal to minimize neonatal failure. Maternal colostrum provides high amounts of nutrient as well as functional compounds that promote immune system and intestinal maturation. Immunoglobulin transfer plays a pivotal role for puppies’ and kittens’ survival, and should be completed by the first 16 h of life. Poor colostrum quality or its inadequate production require holistic and rapid interventions to support newborns. Colostrum deficiencies can lead to severe issues for newborns such as cardiovascular problems, hypothermia, hypoxia, acidosis, anoxia, and hypoglycemia. Different strategies have been proposed to support puppies and kittens in the case of colostrum deficiencies with encouraging results. The set-up of a colostrum bank, the use of bovine colostrum, plasma, and hyperimmune egg powder have been shown to improve puppies’ and kittens’ health. Colostrum is the first source of nutrients for the newborn, and it fulfills all nutrient requirements while also providing several bioactive compounds fundamental for puppies’ and kittens’ long-term development.

## Figures and Tables

**Table 1:** Scheme of colostrum administration and energy requirements of puppies and kittens.

Species	Stomach Capacity	Calories Needs	Calories of Colostrum	Feeding Frequency	References
Kitten	40–50 mL/kg	240–275 kcal/kg	1170 kcal/L	Every 4–6 h	[20,21,22]
Puppy	40–50 mL/kg	130–220 kcal/kg	1800 kcal/L	Every 3–6 h	[1,23]

**Table 2:** Concentrations of nutrients throughout the first week of bitch and queen lactation [22,23].

Nutrient	Bitch Lactation			Queen Lactation		
Composition	Day 1	Day 3	Day 7	Day 1	Day 3	Day 7
True protein (g/L)	143.00 ± 19.20	102.30 ± 13.20	81.70 ± 8.80	83.00 ± 13.00	54.00 ± 3.00	63.00 ± 4.00
NPN (g/L)	1.19 ± 0.05	1.07 ± 0.02	1.15 ± 0.05	0.91 ± 0.12	0.78 ± 0.04	0.81 ± 0.03
NPN (%/N)	5.70 ± 0.70	6.70 ± 0.50	9.10 ± 1.20	7.20 ± 1.20	8.40 ± 0.80	7.50 ± 0.40
Casein (% total protein)	60.70 ± 4.10	75.4.00 ± 2.70	68.20 ± 3.70	40.40 ± 3.80	49.00 ± 0.60	50.40 ± 1.60
Whey (% total protein)	39.30 ± 4.10	24.60 ± 2.70	31.80 ± 3.70	59.60 ± 3.80	51.00 ± 0.06	49.60 ± 1.60
Lipid (g/L)	132.20 ± 16.70	137.20 ± 11.70	132.10 ± 8.30	93.00 ± 19.00	53.00 ± 9.00	76.00 ± 14.00
Lactose (g/L)	16.60 ± 1.30	29.30 ± 2.30	35.40 ± 1.00	29.90 ± 3.40	40.30 ± 1.50	39.00 ± 1.60
Citrate (mM)	4.80 ± 0.40	5.30 ± 0.24	6.60 ± 0.34	6.50 ± 0.30	5.80 ± 0.50	3.90 ± 0.40
Iron (mg/L)	3.70 ± 0.29	6.90 ± 0.57	5.70 ± 0.39	1.85 ± 0.300	3.90 ± 0.55	3.19 ± 0.26
Copper (mg/L)	1.30 ± 0.66	1.40 ± 0.22	1.00 ± 0.13	0.36 ± 0.07	1.34 ± 0.25	1.28 ± 0.16
Zinc (mg/L)	5.00 ± 1.30	5.40 ± 1.00	5.80 ± 0.40	5.78 ± 0.76	6.77 ± 1.24	6.49 ± 0.58
Magnesium (mg/L)	128.50 ± 17.80	85.80 ± 4.90	95.60 ± 5.30	111.00 ± 12.00	82.00 ± 6.00	79.00 ± 7.00
Calcium (mg/L)	1363.00 ± 108.00	1366.00 ± 118.00	1773.00 ± 128.00	462.00 ± 57.00	1162.00 ± 110.00	1586.00 ± 122.00
Phosphorus (mg/L)	935.00 ± 83.00	914.00 ± 162.00	1166.00 ± 136.00	1137.00 ± 45.00	1305.00 ± 55.00	1529.00 ± 57.00
Ca:P	1.50	1.50	1.50	0.10	0.89	1.40
Energy (kcal/L)	1831.00 ± 506.00	1761.00 ± 282.00	1657.00 ± 292.00	1287.00 ± 141.00	853.00 ± 80.00	1093.00 ± 137.00
**Amino Acids**	**Bitch (mmol/L)**			**Queen (mmol/g protein)**		
**Composition**	**Day 1**	**Day 3**	**Day 7**	**Day 1**	**Day 3**	**Day 7**
Alanine	0.170 ± 0.012	0.052 ± 0.007	0.082 ± 0.010	0.539 ± 0.006	0.571 ± 0.010	0.530 ± 0.008
Arginine	0.104 ± 0.007	0.032 ± 0.004	0.056 ± 0.008	0.352 ± 0.011	0.400 ± 0.004	0.400 ± 0.003
Asparagine + Aspartic acid	0.235 ± 0.015	0.073 ± 0.009	0.117 ± 0.014	0.691 ± 0.014	0.799 ± 0.011	0.769 ± 0.010
Cysteine	0.058 ± 0.004	0.021 ± 0.003	0.032 ± 0.005	0.130 ± 0.004	0.149 ± 0.005	0.122 ± 0.004
Glutamine + Glutamic acid	0.426 ± 0.021	0.124 ± 0.018	0.242 ± 0.004	1.474 ± 0.013	1.516 ± 0.015	1.581 ± 0.013
Glycine	0.079 ± 0.008	0.020 ± 0.003	0.027 ± 0.003	0.241 ± 0.016	0.222 ± 0.011	0.198 ± 0.008
Histidine	0.076 ± 0.004	0.021 ± 0.003	0.041 ± 0.007	0.193 ± 0.009	0.198 ± 0.010	0.206 ± 0.008
Isoleucine	0.128 ± 0.007	0.037 ± 0.005	0.069 ± 0.011	0.290 ± 0.008	0.277 ± 0.007	0.291 ± 0.006
Leucine	0.301 ± 0.017	0.093 ± 0.014	0.169 ± 0.026	0.988 ± 0.008	0.972 ± 0.004	0.997 ± 0.006
Lysine	0.128 ± 0.008	0.037 ± 0.005	0.061 ± 0.008	0.491 ± 0.019	0.526 ± 0.016	0.516 ± 0.017
Methionine	0.073 ± 0.005	0.022 ± 0.003	0.036 ± 0.005	0.213 ± 0.007	0.225 ± 0.003	0.206 ± 0.016
Phenylalanine	0.096 ± 0.006	0.027 ± 0.004	0.048 ± 0.007	0.251 ± 0.029	0.234 ± 0.003	0.224 ± 0.002
Proline	0.302 ± 0.017	0.083 ± 0.013	0.168 ± 0.028	1.073 ± 0.011	0.910 ± 0.020	0.929 ± 0.007
Serine	0.138 ± 0.007	0.036 ± 0.005	0.065 ± 0.009	0.545 ± 0.026	0.482 ± 0.006	0.467 ± 0.006
Threonine	0.149 ± 0.009	0.045 ± 0.006	0.070 ± 0.007	0.517 ± 0.011	0.479 ± 0.008	0.480 ± 0.019
Tryptophan	0.007 ± 0.002	n.d.	0.005 ± n.d.	0.094 ± 0.004	0.095 ± 0.007	0.094 ± 0.002
Tyrosine	0.068 ± 0.005	0.021 ± 0.005	0.031 ± 0.004	0.278 ± 0.003	0.274 ± 0.001	0.275 ± 0.001
Valine	0.206 ± 0.013	0.060 ± 0.008	0.102 ± 0.014	0.415 ± 0.014	0.365 ± 0.012	0.376 ± 0.010

NPN: non protein nitrogen; n.d.: not detectable. Data are expressed as means ± standard error.

**Table 3:** Bioactive compounds and growth factors of colostrum.

Bioactive Compounds	Function	Reference
ß-lactalbulin	Potential antiviral, prevention of pathogen adhesion anticarcinogenic and hypocholesterolemic, and hydrophobic components binding ability, including retinol and long-chain fatty acids.	[55]
α-lactalbulin	Calcium metalloprotein, in which Ca plays a crucial role in the folding and structure. Effector of lactose synthesis in mammary gland, calcium carrier, immunomodulatory, precursor for bioactive peptides potentially anticarcinogenic.	[56]
Lactoferrin	Antimicrobial, antioxidative, anticarcinogenic, anti-inflammatory, iron transport, cell growth regulation, precursor for bioactive peptides, immunomodulatory, stimulation of osteoblast proliferation.	[57]
Lactoperoxidase	Antimicrobial, synergistic effects with immunoglobulins, lactoferrin, and lysozyme.	[53]
Lysozyme	Antimicrobial, synergistic effects with immunoglobulins, lactoferrin, and lactoperoxidase.	[58]
**Growth factors**	**Function**	**Reference**
Epidermal growth factor (EGF)	Stimulation of cell growth, intestinal cell protection and repair, regulation of immune system.	[59]
Binding proteins (IGFBP)	Marked anabolic characteristics, gastrointestinal maturation, and wound healing contribution.	[60]
Transforming growth factor -alpha (TGF-α) and beta (TGF-ß)	Cellular proliferation and tissue growth, maturation, and repair activation. Inductive effect on IgA production in Peyer’s patch lymphocytes and spleen lymphocytes.	[61]
Insulin-like growth factor 1 (IGF-1).	Maintenance of adult muscle mass depending on satellite cells activation, proliferation, survival, and differentiation, processes. Lean muscle growth and beta oxidation of fats.	[62]
Hepatocyte growth factor (HGF).	Produced by macrophages, important factor for intestinal cells growth in neonates after birth. DNA and proteins synthesis and nutrient uptake enhancement, particularly in muscles and cartilages.	[63]
Platelet derived growth factor (PDGF)	Gastrointestinal development and maturation.	[64]
Vascular endothelial growth factor vascular (VEGF)	Gastrointestinal growth and perivascular maturation.	[65]
Growth hormone (GH)	Maturation of gastrointestinal mucous membrane development and closing of antibody transport at intestinal level.	[66]

## Data Availability

All presented data are available within the article.
